# High-speed and Large-scale Privacy Amplification Scheme for Quantum Key Distribution

**DOI:** 10.1038/s41598-019-50290-1

**Published:** 2019-10-31

**Authors:** Bang-Ying Tang, Bo Liu, Yong-Ping Zhai, Chun-Qing Wu, Wan-Rong Yu

**Affiliations:** 1 College of Computer, National University of Defense Technology, Changsha, 410073 China; 2 College of Advanced Interdisciplinary Studies, National University of Defense Technology, Changsha, 410073 China; 3 Cyberspace Institute of Advanced Technology, Guangzhou University, Guangzhou, 510006 China

**Keywords:** Quantum information, Information technology

## Abstract

State-of-art quantum key distribution (QKD) systems are performed with several GHz pulse rates, meanwhile privacy amplification (PA) with large scale inputs has to be performed to generate the final secure keys with quantified security. In this paper, we propose a fast Fourier transform (FFT) enhanced high-speed and large-scale (HiLS) PA scheme on commercial CPU platform without increasing dedicated computational devices. The long input weak secure key is divided into many blocks and the random seed for constructing Toeplitz matrix is shuffled to multiple sub-sequences respectively, then PA procedures are parallel implemented for all sub-key blocks with correlated sub-sequences, afterwards, the outcomes are merged as the final secure key. When the input scale is 128 Mb, our proposed HiLS PA scheme reaches 71.16 Mbps, 54.08 Mbps and 39.15 Mbps with the compression ratio equals to 0.125, 0.25 and 0.375 respectively, resulting achievable secure key generation rates close to the asymptotic limit. HiLS PA scheme can be applied to 10 GHz QKD systems with even larger input scales and the evaluated throughput is around 32.49 Mbps with the compression ratio equals to 0.125 and the input scale of 1 Gb, which is ten times larger than the previous works for QKD systems. Furthermore, with the limited computational resources, the achieved throughput of HiLS PA scheme is 0.44 Mbps with the compression ratio equals to 0.125, when the input scale equals up to 128 Gb. In theory, the PA of the randomness extraction in quantum random number generation (QRNG) is same as the PA procedure in QKD, and our work can also be efficiently performed in high-speed QRNG.

## Introduction

Quantum Key Distribution (QKD), which based on the fundamental quantum mechanics, can generate the information-theoretical secure (ITS) keys for distant communication parties^1–3^. Practical QKD systems are mainly composed of two phases: the quantum communication phase and the post-processing phase^4,5^. In the post-processing phase, partial information about the secure key may still be leaked to the eavesdropper Eve after the key/basis sifting and error correction procedures. Privacy amplification (PA), the most significant post-processing procedure, coverts the weak secure correlated key to a uniform and ITS key to Eve^6–8^.

Given the input weak secure key *W* with length of *n* and the security level *ε*, the optimal PA scheme in theory can be achieved with (dual) universal hash functions using Toeplitz kind of matrix (*T*) with computational complexity of *O*(*n*log*n*)^9^, and the length of consumed random seed in PA is *αn*, with min-entropy of *αn* + *O*(1), *α* ∈ (0,1]^10–13^.

Nowadays, state-of-art academic QKD experiments are performed with several GHz pulse rates^14–18^, advanced multiplexing technologies^19,20^ and extracts secure keys even with high-dimensional scenarios^21–23^. Meanwhile, a rigorous statistical fluctuation analysis has to be performed to remove the finite-size key effects on the final secure key^24,25^. Therefore, a high throughput and large-scale (usually larger than several Megabits) PA scheme has to be implemented to real-time extract the secure key with achievable generation rate close to the asymptotic (infinite-key) limit.

The simplest implementation idea of a large-scaled PA scheme is directly performing multiplication operation between *W* and *T*, resulting in the computational complexity with *O*(*n*^2^). However, such matrix-vector multiplication is very suitable to be implemented with Field-Programmable Gate Array (FPGA) platform. H. Zhang *et al*. firstly divided *T* into many smaller blocks and proposed a block parallel PA scheme to speedup the Toeplitz hashing procedure^26^. S. Yang *et al*.^27^ and J. Constantin *et al*.^28^ proposed advanced block partition strategies to reduce the overhead of multiplication operations respectively, resulting in the throughput around 64 Mbps with input scale of 1 megabits^27^.

Actually, majority optimized PA schemes are performed using fast Fourier transform (FFT) with complexity reduced to *O*(*n*log*n*)^8,29,30^. Given fixed security level *ε* (i.e. 10^−10^), the farther communication distance, the larger input length of PA scheme should be adapted. For example, in entanglement-based QKD systems, the input length *n* should be increased to at least the order of 10^8^. B. Liu *et al*. firstly improved the throughput of FFT enhanced PA scheme to 60 Mbps with input scale of 12.8 megabits on Many-Integrated-Core (MIC) platform^8^. Z. L. Yuan *et al*. implemented a number theoretical transform (NTT) based PA scheme with throughput up to 108.77 Mbps with the input scale of 100 megabits also on MIC platform^31^. X. Wang *et al*. proposed a parallel implementation of the length-compatible (up to 10 Gbits) FFT based PA algorithm for continuous-variable QKD systems on a graphic processing unit (GPU) platform, with speed over 1 Gbps^29^.

It’s a huge challenge to implement large-scale FFT based PA schemes on FPGA platforms due to the limited resources and ultra complicated hardware design. Implementation of PA schemes on MIC, GPU or other dedicated computational devices consumes ultra high power and volume and significantly increases the design complexity. Improving the throughput of FFT enhanced PA schemes on CPU platforms is a very conventional option, since it can be efficiently integrated to the whole QKD system. However, it’s feasible with CPU implementations only for small input scales (≤10^6^) and rapidly becomes the performance bottleneck with larger input scales. Therefore, in this article, we propose a fast Fourier transform (FFT) enhanced high-speed and large-scale (HiLS) PA scheme on commercial multi-core CPU platform. In the HiLS PA scheme, *W* is divided into many blocks and the random seed for constructing Toeplitz matrix *T* is shuffled to multiple sub-sequences respectively, then PA procedures are parallel implemented for all sub-key blocks with correlated sub-sequences, afterwards the outcomes are merged as the final secure key. When the input scale is 128 Mb, our HiLS PA scheme reaches 71.16 Mbps, 54.08 Mbps and 39.15 Mbps with the compression ratio equals to 0.125, 0.25 and 0.375 respectively. Therefore, HiLS PA scheme can be applied to 10 GHz QKD systems with even larger input scales and the evaluated throughput is around 32.49 Mbps with the compression ratio equals to 0.125 and the input scale of 1 Gb, which is ten times larger than the previous works for QKD systems. Furthermore, with the limited computational resources (128 GB memory, 1 TB storage and 16 CPU cores in total), the achieved throughput of HiLS PA scheme is 0.44 Mbps with the compression ratio equals to 0.125, when the input scale equals up to 128 Gb. In theory, the PA of the randomness extraction in quantum random number generation (QRNG) is same as the PA procedure in QKD^32–34^. Thus, HiLS PA scheme can also be efficiently performed in high-speed QRNG.

## Related Work

**Figure 1:**
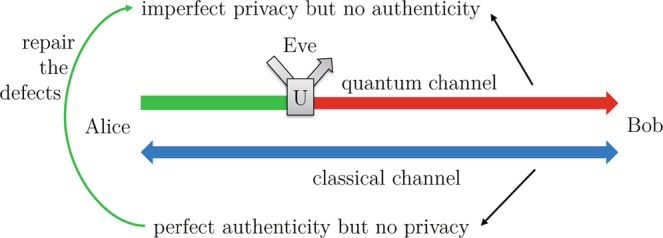
Schematic diagram of privacy amplification in quantum key distribution.

Privacy amplification was first proposed in the context of quantum key distribution by Bennett *et al*.^6^, where the channel with perfect authenticity but no privacy (public classical channel) can be used to repair the defects of a channel with imperfect privacy but no authenticity (quantum channel). The schematic diagram of PA in QKD is shown in Fig. 1, Alice and Bob firstly distribute quantum signals via a noisy and lossy quantum channel (fiber or free space), then share correlated and weak secure key *W* after basis/key sifting and error correction procedures via a public channel. The min-entropy of shared weak secure key *W* is *n*. Let random variable *E* summarizes Eve’s entire learned knowledge about *W*, here, *H*(*W*|*E*) ≤ *t*, *t* < *n*. PA, where Alice and Bob publicly discuss a extractor function *G*:{0,1}^*n*^→{0,1}^*r*^, such that reduces Eve’s learned information of the final secure key *K*_*f*_ from *t* to at most *ε*^6,7,35,36^. Nowadays, most practical extractors are known to the universal hash function, especially the (modified) Toeplitz matrix defined as^13^

1 $$G(A)\,:=({I}_{r}|T(A))=[\begin{array}{ccccccccc}1 &  &  &  &  & {a}_{r-1} & {a}_{r} & \ldots  & {a}_{n-2}\\  & 1 &  &  &  & {a}_{r-2} & {a}_{r-1} & \ldots  & {a}_{n-3}\\  &  & \ddots  &  &  & \vdots  & \vdots  & \ddots  & \vdots \\  &  &  &  & 1 & {a}_{0} & {a}_{1} & \cdots  & {a}_{n-r-1}\end{array}],$$

where *T*(*A*) is a *r* × (*n* − *r*) Toeplitz matrix, *A* is a random seed, *A* = (*a*_0_, *a*_1_, …, *a*_*n*−1_) ∈ {0,1}^*n*−1^, *T*(*A*)_*i*,*j*_ = *a*_*j*−*i*+*r*−1_. Also, we define *W*_I_ = (*w*_0_, *w*_1_, …, *w*_*r*−1_) and *W*_TA_ = (*w*_*r*_, *w*_*r*+1_, …, *w*_*n*−1_). Therefore, the final secure key can be calculated as

2 $${K}_{f}=G(A)W={I}_{r}\times ({w}_{0},{w}_{1},\ldots ,{w}_{r-1})\oplus T(A)\times ({w}_{r},{w}_{r+1},\ldots ,{w}_{n-1})={W}_{{\rm{I}}}\oplus T(A){W}_{{\rm{TA}}}.$$

In order to efficiently implement the calculation of *T*(*A*)*W*_TA_ using fast Fourier transform (FFT), we have to extend *T*(*A*) to a special circulant Toeplitz matrix with scale of (*n* − 1) × (*n* − 1) and extend *W*_TA_ to a vector with length of *n* − 1 by padding zeros. The optimized multiplication of a circulant matrix and a vector is shown as

3 $$H\cdot X={F}^{-1}[F(h)\,\ast \,F(X)],$$

where “*” denotes the Hadamard product operator, *F* denotes the Fourier transform operator, *F*^−1^ is the inverse Fourier transform operator, *X* is a vector and *H* is a circulant Toeplitz matrix with first row *h*. Since the complexity of *F* and *F*^−1^ operations is *O*(*n*log*n*) and the complexity of Hadmard product operation is *O*(*n*), the computational complexity of optimized PA algorithm is *O*(*n*log*n*)^8,12^.

In theory, QKD can generate ITS keys for communication parties, even the quantum channel is under control of the eavesdropper Eve. Imperfect implementation and active attacks would leak some information about *W* to Eve. Alice and Bob can quantify the bound of leaked information accurately with the infinite post-processing block size. In this paper, we take entanglement based QKD as an example, the secure key rate can be calculated as^37^

4 $$R\ge q{Q}_{\mu }{\nu }_{s}[1-{H}_{2}({e}_{p}^{U})-f({e}_{b}){H}_{2}({e}_{b})],$$

where *q* is the basis sifting factor, *Q*_*μ*_ is the gain of detected entangled photon pairs, *ν*_*s*_ is the repetition rate of the entangled source, *e*_*b*_ is the measured quantum bit error rate, ${e}_{p}^{U}$ is the estimated upper-bound of phase error rate, *f*(*x*) is the error correction efficiency, *H*_2_(*x*) is the binary Shannon entropy.

**Figure 2:**
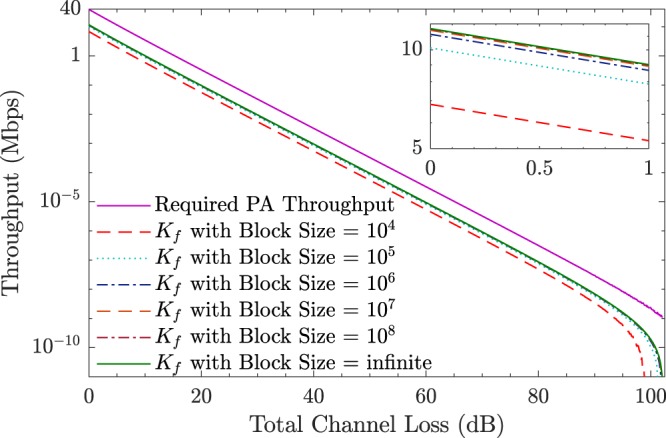
Required throughput of PA algorithms and final secure key rate with different block sizes for 10 GHz entanglement based QKD systems, under the simulation parameters shown in Table 1.

In practice, *e*_*p*_^*U*^ can not be measured directly and could not be accurately estimated due to the statistical fluctuations with finite post-processing block sizes. Here, we simulate the required throughput of PA algorithm in a 10 GHz entanglement based QKD with the parameters shown in Table 1. The entangled photon source is put into the middle of communication parties, the finite-size-effect for the final secure key *K*_*f*_ is considered with post-processing block size from the order of 10^4^ to infinite, and the failure probability *ε*^*ph*^ = 10^−10^ for estimating ${e}_{p}^{U}$^4^. The analyzed results are shown in Fig. 2, the post-processing block size should be at least the order of 10^8^ to achieve a secure key rate close to the asymptotic limit. Directly implementing PA algorithms with ultra large-scale inputs will limit the performance of full QKD systems. Meanwhile, the required throughput of PA algorithm is around 40 Mbps without any channel loss.

**Table 1:** Parameters used for simulation of entanglement based QKD.

Parameter	Values
Pulse Repetition Rate *ν*_*s*_	10 GHz
Heralding Efficiency	0.316
Dark Count Rate *p*_*d*_	10^−7^
Detector Efficiency *η*_*d*_	0.40
Misalignment Error Rate *e*_*d*_	0.015
Error Correction Efficiency *f*	1.10
Photon Pair Number per Coincidence Window *μ*	Optimal
Basis Reconciliation Factor *q*	0.50
Phase Error Estimation Failure Probability *ε*^*ph*^	10^−10^

## High-speed and Large-scale Privacy Amplification Scheme

**Figure 3:**
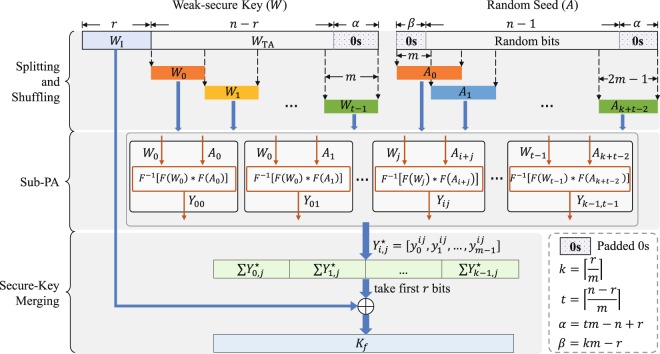
Schematic diagram of proposed high-speed and large-scale privacy amplification scheme for QKD. The weak secure key length is *n*, the final secure key length is *r*, the sub-block size is *m*, 0 < *m* ≤ *r* < *n*, $t=\lceil \frac{n-r}{m}\rceil$, $k=\lceil \frac{r}{m}\rceil$. *Y*_*i*,*j*_ = *F*^−1^[*F*(*A*_*i*+*j*_) * *F*(*W*_*j*_)], where “*” denotes the Hadamard product operator, *F* denotes the Fourier transform operator, *F*^−1^ is the inverse Fourier transform operator. ${Y}_{i,j}^{{\rm{a}}}$ is a sub-vector consisted by first *m* bits of *Y*_*i*,*j*_, defined as ${Y}_{i,j}^{{\rm{a}}}=[{y}_{0}^{ij},{y}_{1}^{ij},\ldots ,{y}_{m-1}^{ij}]$.

The schematic diagram of proposed high-speed and large-scale (HiLS) privacy amplification scheme for QKD is shown in Fig. 3. Weak secure key *W* with length of *n* is gained after the basis/key sifting and error correction procedures for the measured raw key string at Alice’s (Bob’s) side. Then, Alice and Bob estimate the final secure key length *r* with rigorous statistical fluctuation analysis procedure. Afterwards, Alice and Bob publicly discuss a random seed with length of *n* − 1 bits to construct the universal hash function. Our proposed HiLS PA scheme mainly consists of three steps: splitting and shuffling, sub-PA and secure-key merging.

### Step 1: Splitting and shuffling

In this step, we divide *W* to several sub-vectors and divide the Toeplitz matrix *T*(*A*) to sub-matrices. Assume the scale of sub-matrix is *m* × *m*, *m* ≤ *r*. Assume that the Toeplitz matrix *T*(*A*) can be divided into *t* blocks by rows and *k* blocks by columns, thus in total *kt* sub-matrices, $t=\lceil \frac{n-r}{m}\rceil$, $k=\lceil \frac{r}{m}\rceil$. First of all, we construct a vector *A* by padding *km* − *r* (*tm* − *n* + *r*) zeros to the head (tail) of the exchanged random seed with length of *n* − 1 bits. Then, we shuffle *A* into *k* + *t* − 1 sub-vectors, defined as *A*_*i*_: = [*a*_*im*_, *a*_*im *_+_ 1_, …, *a*_(2+*i*)*m*−1_], 0 ≤ *i* < *k* + *t* − 1. Therefore, the divided sub-matrix can be constructed by *H*_*i*,*j*_ = *T*(*A*_*i*+*j*_), *i* ∈ [0, *k*) and *j* ∈ [0, *t*), and we have

5 $$T(A)=[\begin{array}{cccc}{H}_{k-1,0} & {H}_{k-1,1} & \cdots  & {H}_{k-1,t-1}\\ {H}_{k-2,0} & {H}_{k-2,1} & \cdots  & {H}_{k-2,t-1}\\ \vdots  & \vdots  & \ddots  & \vdots \\ {H}_{00} & {H}_{01} & \cdots  & {H}_{0,t-1}\end{array}]=[\begin{array}{cccc}T({A}_{k-1}) & T({A}_{k}) & \cdots  & T({A}_{k+t-2})\\ T({A}_{k-2}) & T({A}_{k-1}) & \cdots  & T({A}_{k+t-3})\\ \vdots  & \vdots  & \ddots  & \vdots \\ T({A}_{0}) & T({A}_{1}) & \cdots  & T({A}_{t-1})\end{array}],$$

where *H*_*i*,*j*_ = *H*_*i*+1, *j*+1_.

For *W*, we first pad *tm* − *n* + *r* zeros to the tail and take first *r* bits and the rest bits to construct the sub-vector *W*_I_ and *W*_TA_. Then, divide *W*_TA_ into *t* sub-vectors, defined as *W*_*i*_: = [*w*_*im*+*r*_, *w*_*im*+*r*+1_, …, *w*_(*i*+1)*m*+*r*−1_], where 0 ≤ *i* < *t*.

### Step 2: Sub-PA

In this step, the efficient implementation using FFT of multiplication *Y*_*i*,*j*_ is performed to sub-vector *W*_*j*_ and sub-matrix *H*_*i*,*j*_,

6 $${Y}_{i,j}\,:={F}^{-1}[F({A}_{i+j})\,\ast \,F({W}_{j})],$$

where, *i* ∈ [0, *k*) and *j* ∈ [0, *t*).

### Step 3: Secure-Key merging

First, we only take first *m* bits of *Y*_*i*,*j*_ (defined as ${Y}_{i,j}^{{\rm{a}}}$), then we merge ${Y}_{i,j}^{{\rm{a}}}$ to vector *Z* by

7 $$Z=({\sum }^{t-1}\,{{\rm{y}}}_{0,j}^{\ast }|{\sum }^{t-1}\,{{\rm{y}}}_{1,j}^{\ast }|\ldots |{\sum }^{t-1}\,{Y}_{k-1,j}^{\ast }).$$

Take first *r* bits of *Z* (defined as Z^*^), we can get the final secure key *K*_*f*_ by

8 $${K}_{f}={W}_{{\rm{I}}}\oplus {Z}^{\ast }.$$

The detailed implementation of HiLS PA scheme can be described as Algorithm 1. In the procedure of our proposed HiLS PA scheme, we only need to perform *k* + 2*t* − 1 times Fourier operations with scale of 2*m*, *kt* times Hadamard product operations with scale of *m*, *kt* times inverse Fourier operations and *kt* + 1 times exclusive or (XOR) operations with scale of *m*. Thus, the computational complexity of the proposed HiLS PA scheme is *O*(*ktm*log*m*), simplified to around *O*(*n*log*m*).

**Algorithm 1:**
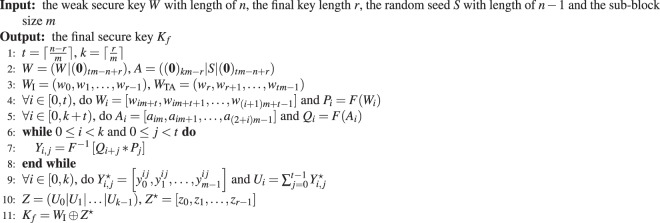
HiLS Privacy Amplification algorithm.

## Results

The implementation of HiLS PA scheme is evaluated on the multi-core server computer, the specifications are shown in Table 2. Due to FFT operation may suffer errors caused by finite-precision float-point arithmetic, we suggest the scale of FFT operation smaller than the order of 10^8^. Meanwhile, considering the thread synchronization and thread safety issues, the calculations of (inverse) Fourier transforms and also Hadamard products are paralleled in the architecture of shared memory multi-processes.

**Table 2:** Specifications of server computer.

Parameter	Values
Operation System	CentOS 7
CPU	Intel(R) E5-2640 v3 × 2
Cores per CPU	8
Threads per core	2
Memory	128 GB
Storage	1 TB
Compiler	gcc 4.8.5
MPI	openmpi 1.10.7
FFT library	fftw 3.3.8

**Figure 4:**
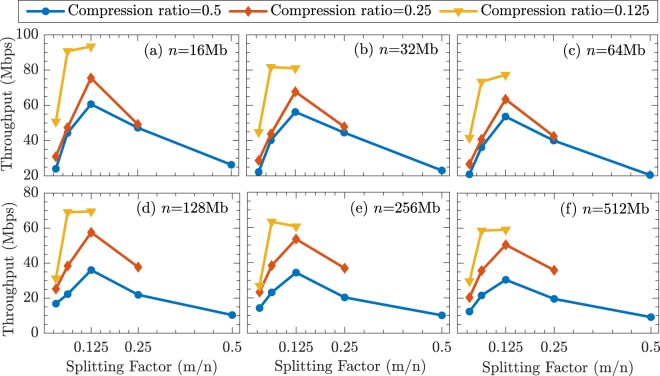
Throughput of HiLS PA scheme with *n* equals from 16 Mb to 512 Mb, and the splitting factor $\frac{m}{n}$ varies from $\frac{1}{32}$ to $\frac{1}{2}$.

We evaluate the throughput of HiLS PA scheme with different input scale (*n*) and various sub-block size (*m*). The result is shown in Fig. 4, where we set the input weak secure key length *n* equals from 16 Mb to 512 Mb, and splitting factor, defined as $\frac{m}{n}$ is various from $\frac{1}{32}$ to $\frac{1}{2}$. Figure 4 shows us that for given *n* (in our implementation, can be up to 1 Gb), HiLS PA scheme can always achieve optimized throughput when splitting factor $\frac{m}{n}=0.125$. When the splitting factor $\frac{m}{n}\le 0.0625$, the Toeplitz matrix at least has to be divided into 28 sub-matrices with compression ratio $\frac{r}{n}\ge 0.125$, larger than 16 (amount of total cores), resulting HiLS PA scheme with very poor throughput due to heavy overhead of complicated process scheduling. When the splitting factor $\frac{m}{n}\ge 0.25$, less split sub-matrices (≤4) only contributes a bit speedup to HiLS PA scheme, due to not fully used computational resource and still large scaled FFT operations. When the splitting factor $0.125 < \frac{m}{n} < 0.25$, the amount of split sub-matrices stays the level as the case with splitting factor equals to 0.125, but the FFT operating scale is same as the case with splitting factor equals to 0.25, which results even worse throughput to HiLS PA scheme. This situation would also happened when the splitting factor $0.0625 < \frac{m}{n} < 0.125$. For example, when *n* = 512 Mb, the optimized throughput of HiLS PA scheme is 59.06 Mbps, 50.48 Mbps and 30.49 Mbps when the compression ratio equals to 0.125, 0.25 and 0.50 respectively.

**Figure 5:**
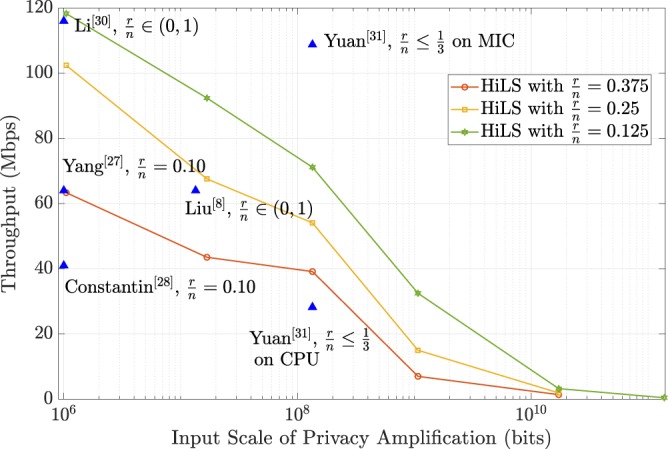
Comparison of HiLS PA scheme and privous works.

According to the simulation results shown in Fig. 2, the maximum compression ratio required for PA schemes is ($\frac{r}{n}$) is 0.297 for 10 GHz entanglement based QKD systems. Then, we optimized the implementation of HiLS PA scheme with *n* = 1 Mb, 16 Mb, 128 Mb and 1 Gb with compression ratio equals to 0.125, 0.25 and 0.375 respectively and compared with other previous works designed for QKD systems, e.g. entanglement based systems, the results are shown in Fig. 5. S. Yang *et al*.^27^ and J. Constantin *et al*.^28^ both implemented PA schemes on FGPA platform by performing multiplication operations, achieved 64.0 Mbps and 41.0 Mbps throughput with compression ratio equals to 0.10. Q. Li *et al*. achieved the throughput of 116.0 Mbps with adaptive compression ratio by implementing FFT operation on FPGA platform^30^. However, FPGA platform is not suitable for the implementation of PA schemes with ultra-large input scales (larger than the order of 10^8^). B. Liu *et al*. achieved the throughput of 60 Mbps with input scale of 12.8 megabits by implementing the FFT enhanced PA scheme on MIC platform^8^. Z. L. Yuan *et al*. achieved the throughput of 108.77 Mbps with the input scale supported up to 128 megabits by implementing the NTT based PA scheme on MIC platform^31^. Z. L. Yuan *et al*. also evaluated the performance of their PA scheme on CPU platform, resulting in the throughput of 28.22 Mbps. When the input scale is 128 Mb, the finite-size-effect for the final secure key can be almost perfectly avoided, and the throughput of our proposed HiLS PA scheme reaches up to 71.16 Mbps, 54.08 Mbps and 39.15 Mbps with the compression ratio equals to 0.125, 0.25 and 0.375 respectively. In the case of input scale is 1 Gb, the throughput of HiLS PA scheme reaches up to 32.49 Mbps and 15.0 Mbps with the compression ratio equals to 0.125 and 0.25, which contributes much rigorous statistical fluctuation analysis and is remarkable higher than the required throughput when the total channel loss is expected larger than 87.6 dB.

With limited resource (128 GB memory, 1 TB storage and 16 CPU cores in total), the HiLS PA scheme with input scale of 128 Gb and the compression ratio equals to 0.125, runs around 83 hours, resulting a throughput of 0.44 Mbps. The implementation of PA with such large inputs on GPU platform is very difficult due to the complicated computation and memory scheduling strategies. Meanwhile, the throughput of the HiLS PA scheme can be easily improved on high-speed multi-core CPU platforms with much larger configured memory.

## Conclusion

In this paper, we propose a fast Fourier transform (FFT) enhanced high-speed and large-scale (HiLS) PA scheme on multi-core CPU platform. The long input weak secure key is divided into many blocks and the random seed for constructing Toeplitz matrix is shuffled to multiple sub-sequences respectively, then PA procedures are parallel implemented for all sub-key blocks with correlated sub-sequences, afterwards the outcomes are merged as the final secure key. When the input scale is 128 Mb, our proposed HiLS PA scheme reaches 71.16 Mbps, 54.08 Mbps and 39.15 Mbps with the compression ratio equals to 0.125, 0.25 and 0.375 respectively, resulting achievable secure key generation rates close to the asymptotic limit. HiLS PA scheme can be efficiently implemented on the commercial CPU platform without increasing dedicated computational devices and can be applied to 10 GHz QKD systems with even larger input scales. The evaluated throughput of HiLS PA scheme is around 32.49 Mbps with the compression ratio equals to 0.125 and the input scale of 1 Gb, which is ten times larger than the previous works for QKD systems.Furthermore, with the limited computational resources, the achieved throughput of HiLS PA scheme is 0.44 Mbps with the compression ratio equals to 0.125, when the input scale equals up to 128 Gb. As randomness extraction with Toeplitz hashing in QRNG is particularly efficient, the HiLS PA scheme can be also performed in high-speed QRNG.
